# Bush’s Fluid Food

**Published:** 1889-09

**Authors:** 


BUSH'S FLUID FOOD.—Please examine this preparation as found on page 7 of advertising department.

We have found the Bush Manufacturing Company to be reliable and enterprising gentlemen. As a nutritive preparation their fluid food, or Bovinine, is rich in nitrogenous substances and phosphates, and is highly recommended in all cases of mal-nutrition and mal-assimilation. The profession should avail themselves of this valuable food material in the treatment of low and debilitated conditions.

